# Escape from mitotic catastrophe by actin-dependent nuclear displacement in fission yeast

**DOI:** 10.1016/j.isci.2020.102031

**Published:** 2021-01-01

**Authors:** Masashi Yukawa, Yasuhiro Teratani, Takashi Toda

**Affiliations:** 1 Hiroshima Research Center for Healthy Aging (HiHA), Hiroshima University, Higashi-Hiroshima 739-8530, Japan; 2 Laboratory of Molecular and Chemical Cell Biology, Graduate School of Integrated Sciences for Life, Hiroshima University, Higashi-Hiroshima 739-8530, Japan

**Keywords:** Biological Sciences, Genetics, Molecular Biology, Chromosome Organization, Molecular Genetics, Cell Biology

## Abstract

Eukaryotic cells position the nucleus within the proper intracellular space, thereby safeguarding a variety of cellular processes. In fission yeast, the interphase nucleus is placed in the cell middle in a microtubule-dependent manner. By contrast, how the mitotic nucleus is positioned remains elusive. Here we show that several cell-cycle mutants that arrest in mitosis all displace the nucleus toward one end of the cell. Intriguingly, the actin cytoskeleton is responsible for nuclear movement. Time-lapse live imaging indicates that mitosis-specific F-actin cables possibly push the nucleus through direct interaction with the nuclear envelope, and subsequently actomyosin ring constriction further shifts the nucleus away from the center. This nuclear movement is beneficial, because if the nuclei were retained in the center, unseparated chromosomes would be intersected by the contractile actin ring and the septum, imposing the lethal cut phenotype. Thus, fission yeast escapes from mitotic catastrophe by means of actin-dependent nuclear movement.

## Highlights

• Actin-dependent mitotic nuclear positioning in fission yeast
• Actin cables and ring closure drive nuclear displacement upon mitotic arrest
• Nuclear displacement evades cut-mediated cell death
• Survivors resume cell division as diploids

## Graphical Abstract

**Figure:**
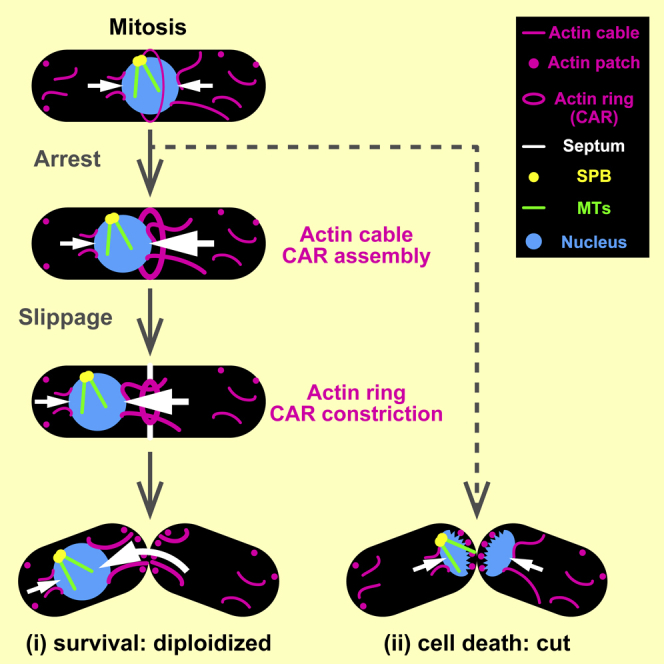

## Introduction

Proper nuclear positioning is essential for the execution of a wide range of cellular processes in eukaryotic cells (Gundersen and Worman, 2013). In non-dividing differentiated cells, the position of the nucleus, which is often coupled with cell shape, is strictly regulated by intracellular forces including tensile and shear stresses or cortical tension. Proper nuclear geometry within these cells plays a vital role in cell motility, cell-cell contact, and developmental decisions (Kopf et al., 2020; Lele et al., 2018).

In proliferating mitotic cells, nuclear positioning is also crucial for successful cell division. The bipolar spindle, which pulls sister chromatids toward two opposite poles, needs to assemble in the geometrical center of the cell. This ensures symmetrical positioning of the two nuclei that are reformed upon mitotic exit. A contractile actomyosin ring (CAR) formed in the middle of the cell then constricts, by which two equal-sized daughter cells inherit the identical set of the chromosomes. Perturbations in this process, such as biased positioning of the nucleus/chromosomes or the CAR, would result in the production of polyploid and anucleate progenies or lead to aneuploidy, potential risk factors for tumorigenesis, and various human diseases (Gordon et al., 2012; Lele et al., 2018; Shu et al., 2019; Umbreit et al., 2020; Yaguchi et al., 2018).

In various cell types, two major cytoskeletal structures, microtubules (MTs) and actin, are integral in positioning the nucleus at the proper geometrical site such as an asymmetric position in polarized cells and the cell middle in dividing cells (Lee and Burke, 2018; Wuhr et al., 2009). In budding yeast, the nucleus that originally locates within the mother cell translocates toward the bud neck in an actin- and MT-dependent manner, thereby ensuring equal partition of the nuclei to each mother and daughter cell (Xiang, 2018). In early embryos of *C. elegans*, pushing forces generated by astral MTs play a pivotal role in spindle centering and movement (Garzon-Coral et al., 2016), whereas in mouse oocytes, the actin cytoskeleton, which forms a meshlike network, is required for centering the nucleus/chromosomes (Colin et al., 2020).

In interphase fission yeast cells, the MT cytoskeleton plays a major role in nuclear positioning; the plus ends of antiparallel MTs that emanate from multiple sites on the nuclear envelope (NE) reach and push the cell tip at either end, centering the nucleus through counteracting forces in a dynamic yet coordinated manner (Daga et al., 2006b; Tran et al., 2001). This regulatory system has provided general insight into the molecular mechanisms by which the nucleus is centered in other types of cells (Wuhr et al., 2009). In contrast, how the nucleus is retained in the center of the cell during mitosis remains elusive. Previous studies showed that a simple centrifugation leads to physical displacement of the mitotic nucleus from the cell center (Carazo-Salas and Nurse, 2006; Daga and Chang, 2005; Daga et al., 2006a), indicating that the mitotic cytosol is plastic and prone to a mechanical force as in interphase.

One condition under which the mitotic nucleus becomes displaced is known for mutations in Pkl1/Kinesin-14 and its cofactors, Msd1 and Wdr8 (Toya et al., 2007; Yukawa et al., 2015). These proteins are required for anchoring the minus end of the spindle MTs to the spindle pole body (SPB, the fungal equivalent of the animal centrosome) and in their absence, spindle MT fails to be tethered to the SPB. Long protruding MTs then reach the cell tip, which in turn produces a pushing force toward the NE, leading to nuclear displacement (Syrovatkina and Tran, 2015). The other condition is simultaneous inactivation of an MT polymerase Dis1 and Klp5/Kinesin-8. In these double mutants, mitotic spindles become extremely elongated, leading to one end of spindles in contact with the cell cortex. This pushing force imposes nuclear displacement (Pinder et al., 2019).

In this study, we have addressed how fission yeast cells locate the nucleus during mitosis. We show that several cell-cycle mutants that arrest in mitosis all displace the nucleus from the cell center. Intriguingly, the actin cytoskeleton, not the MT counterpart, drives this asymmetric geometry of the nucleus. Precisely, mitosis-specific F-actin cables are responsible for nuclear movement, and forces generated through CAR constriction further displace the nucleus from the cell center. These cells subsequently escape from mitotic catastrophe, in which chromosomes would be intersected upon cytokinesis, leading to a lethal cut (Chang et al., 2001; Cullati and Gould, 2019; Hirano et al., 1986). We discuss potential physiological significances of mitotic nuclear displacement.

## Results

### Mitotic arrest leads to nuclear displacement

**Figure 1:**
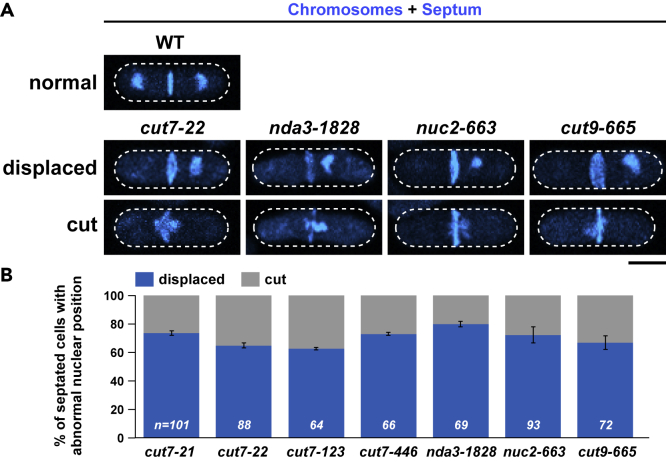
Mitotic arrest leads to nuclear displacement (A) Nuclear displacement in several mitotic mutant cells. Exponentially growing wild-type or indicated mitotic mutants grown at 27°C were shifted to 36°C and incubated for 3 h except for *nda3-1828*, which was incubated for 6 h. Cells were fixed with methanol and stained with DAPI (chromosomes) and Calcofluor (septa). Representative cells for wild-type (top row), mutants displaying displaced chromosomes (middle row), or cut (bottom row) are shown. Scale bar, 5 μm. (B) The percentage of cells showing displaced chromosomes or cut. The sample numbers (n) for individual strains are indicated on the bottom of columns. Data are presented as the means ± SD. See also Figure S1.

Cut7 in fission yeast belongs to the Kinesin-5 family and plays an essential role in bipolar spindle assembly (Hagan and Yanagida, 1990; Yukawa et al., 2020). Its inactivation leads to the emergence of monopolar spindles and mitotic arrest. Revisiting defective phenotypes of *cut7* temperature-sensitive (ts) mutant cells indicated that chromosomes were often displaced from the cell center upon incubation at 36°C, by which septated cells contained one compartment with chromosomes, whereas the other compartment was anucleate (Figure 1A). On the other hand, cells whose nuclei were retained in the middle displayed the “cut” phenotype (Hirano et al., 1986; Yanagida, 1998), in which chromosomes are intersected by the septum.

Nuclear displacement is not specific to Kinesin-5 malfunction, as we observed the same phenotypes in other mitotic mutants including mutations in β-tubulin (*nda3-1828*) (Radcliffe et al., 1998) and two subunits of the Anaphase-Promoting Complex/Cyclosome (APC/C) (*nuc-2663* and *cut9-665*) (Hirano et al., 1988; Samejima and Yanagida, 1994; Yamada et al., 1997) (Figures 1A and 1B). It is of note that the displacement of the chromosomes in *nuc2-663* and *cut9-665* mutants was previously noted (Yanagida, 1998). As fungi undergo a closed mitosis, the displacement of the chromosomes would stem from a defect in nuclear positioning (Figures S1A and S1B). As a representative, we used *cut7-22* for the following investigation, unless otherwise stated.

### The nucleus becomes off-center as the medial actomyosin ring assembles

**Figure 2:**
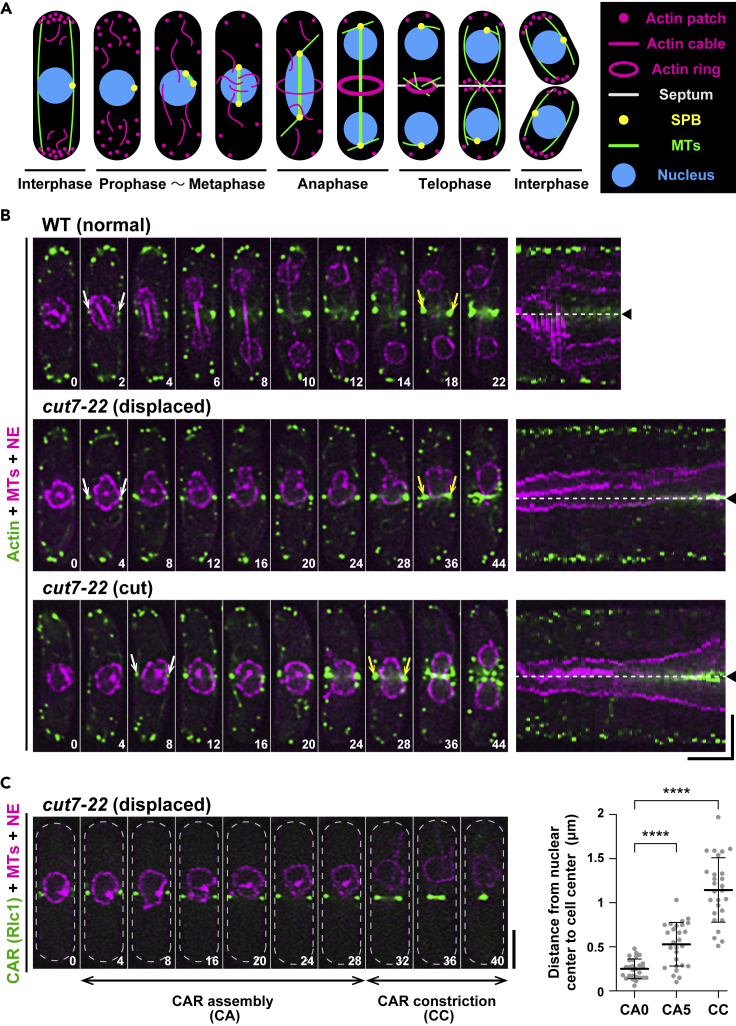
The nucleus becomes off-center as the medial actomyosin ring assembles (A) A schematic depiction of the nucleus and cytoskeletons during the mitotic cell cycle in fission yeast. (B) Time-lapse and kymograph images. Wild-type (top row) or *cut7-22* cells (middle and bottom rows) grown at 27°C were shifted to 36°C and incubated for 2 h, when time-lapse imaging started. Cells contain mCherry-Atb2 (magenta, MTs), Cut11-mRFP (magenta, the NE), and LifeAct-GFP (green, actin). The first time points when LifeAct-GFP signals were observed in the middle of cells are indicated with white arrows, whereas those when the CAR initiated constriction are marked with yellow arrows. Corresponding kymographs are shown on the right (one-minute interval images were merged), in which the middle of the cell axis is shown with dotted lines and arrowheads. Scale bars, 10 min (horizontal) and 5 μm (vertical). See also Videos S1, S2, and S3. (C) Timing of nuclear displacement in relation to that of CAR assembly and constriction. *cut7-22* cells containing mCherry-Atb2 (magenta, MTs), Cut11-mRFP (magenta, the NE), and Rlc1-GFP (green, the CAR) were grown at 27°C and shifted to 36°C for 2 h, when time-lapse imaging started (left, see Video S4). The duration of CAR assembly (CA) and constriction (CC) are marked with horizontal arrow bars. Scale bar, 5 μm. On the right graph, the position of the nucleus (the distance between the center of the cell axis and that of the nucleus) is plotted against initiation of CAR assembly (CA0), 5 min after CAR assembly (CA5), and initiation of CAR constriction (CC). Data are presented as the means ± SD. All p values were obtained from the two-tailed unpaired Student's t test. ∗∗∗∗p < 0.0001. The numbers on the bottom right corner of each image show times in minutes (B and C). See also Figure S2.

We examined the dynamics of nuclear movement using time-lapse fluorescence microscopy. For this purpose, wild-type and *cut7-22* strains were tagged with fluorescent markers for the nuclear envelope (NE, Cut11-mRFP; Cut11 is also localized to the mitotic SPB, West et al., 1998), MTs (MTs, mCherry-Atb2; α2-tubulin, Toda et al., 1984), and actin (LifeAct-GFP, Huang et al., 2012). In fission yeast, the CAR initiates assembly during early mitosis, matures into a complete ring, and then constricts in telophase, followed by cytokinesis (a schematic illustration is shown in Figure 2A and time-lapse images are shown in Figure 2B; see Video S1). Live imaging of *cut7* mutant cells incubated at the restrictive temperature indicated that there were two populations as described earlier (Figure 1B). The first type (30/57) exhibited nuclear displacement (Figure 2B; see Video S2). The second type (27/57) represented ellipsoidal nuclei with the CAR being formed in the middle of the cell axis destined for the cut phenotype (Figure 2B; see Video S3). Interestingly, the timing of nuclear displacement appeared to coincide with or was close to that of CAR assembly.

Video S1. Nuclear positioning in a mitotic wild-type cell, related to figure 2

Video S2. Nuclear displacement in the cut7-22 mutant, related to figure 2

Video S3. A cut7-22 cell displaying cut with the nucleus being retained in the cell center, related to figure 2

To precisely clarify the temporal order between the nuclear movement and CAR assembly, *cut7* cells containing Cut11-mRFP and mCherry-Atb2 were tagged with Type II myosin regulatory light chain (Rlc1-GFP), which is localized to the medial region as a component of the CAR (Le Goff et al., 2000; Naqvi et al., 2000). As shown in Figure 2C (also see Video S4), the nucleus started to be displaced as the CAR assembled before CAR constriction. We addressed whether mitotic delay is necessary for nuclear displacement. For this purpose, the *mad2* gene, encoding a core component of the spindle assembly checkpoint (SAC) (Musacchio, 2015), was deleted in *cut7-22*. The degree of nuclear displacement was lessened; however, we still observed nuclear movement in *cut7-22mad2Δ* cells (Figure S2). Therefore, during prolonged mitotic arrest, the nucleus moves toward one end of the cell as these cells undergo CAR assembly, and yet this could happen without mitotic delay.

Video S4. Timing of the initiation of nuclear displacement, CAR (Rlc1) assembly, and constriction in cut7-22, related to figure 2

### Polymerized actin structures other than endocytic patches drive nuclear movement

**Figure 3:**
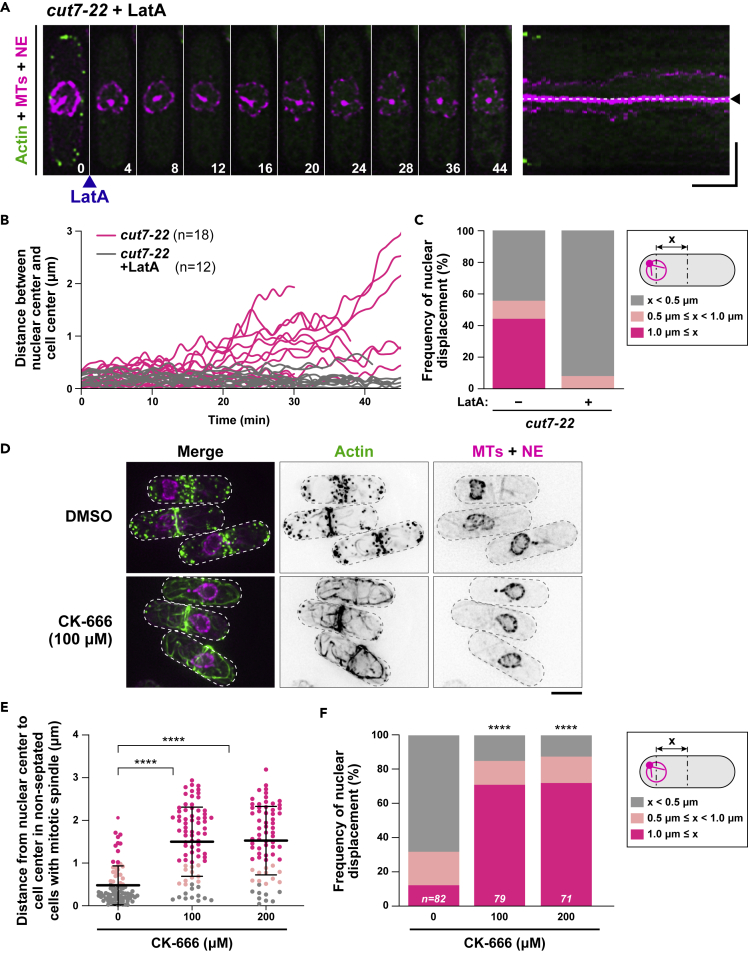
Polymerized actin structures other than endocytic patches drive nuclear movement (A) Time-lapse and kymograph images of the nuclear position in *cut7-22* cells incubated at 36°C in the presence of LatA. *cut7-22* mutant cells grown at 27°C were shifted to 36°C and incubated for 2 h (time 0). At this point, LatA (50 μM) was added and time-lapse imaging started. Cells contain mCherry-Atb2 (magenta, MTs), Cut11-mRFP (magenta, the NE), and LifeAct-GFP (green, actin). Note that actin signals already disappeared 4 min after LatA treatment. The position of the cell center is shown with dotted lines and arrowheads, and the numbers on the bottom right indicate times in minutes upon recording. Scale bars, 10 min (horizontal) and 5 μm (vertical). See also Video S5. (B) Profiles of the relative position of the nucleus in *cut7-22* mutants in the absence (n = 18) or presence (n = 12) of LatA. The distance (x μm) between the center of the cell axis and that of the nucleus was measured at each time point and plotted against time. (C) The degree of nuclear movement in *cut7-22* cells in the absence or presence of LatA. The percentage of cells that do or do not show nuclear displacement is shown. For each sample, the maximal distance (x μm) between the center of the cell axis and that of the nucleus was determined using the data shown in (B) and categorized into three classes: displaced (shown in magenta, in which x is ≥ 1 μm), mildly displaced (shown in pink, in which x is between 0.5 μm and 1 μm), and centered (shown in gray, in which x is ≤ 0.5 μm). See also Figure S3. (D) Lack of actin patches in *cdc7-22* cells treated with CK-666. *cut7-22* cells containing mCherry-Atb2 (MTs, magenta), Cut11-mRFP (the NE, magenta), and LifeAct-GFP (actin, green) were grown at 27°C and shifted to 36°C for 2 h. CK-666 (100 μM) was added, and images were taken 20 min after CK-666 addition. Scale bar, 5 μm. (E) The nuclear position in *cut7-22* cells treated with CK-666. *cut7-22* cells containing mCherry-Atb2 (MTs), Cut11-mRFP (the NE), and LifeAct-GFP (actin) were grown at 27°C and shifted to 36°C for 2 h. CK-666 (100 or 200 μM) or DMSO was added. Samples were observed 20 min after CK-666 addition. The distance (x μm) between the center of the cell axis and that of the nucleus was measured for each cell and plotted in the graph as dots: gray, x < 0.5 μm; pink, 0.5μm < x < 1.0 μm; magenta, x > 1.0 μm. Mitotic cells before CAR constriction were counted. Data are given as mean ± SD. p values were obtained from the two-tailed unpaired Student's t test. ∗∗∗∗p < 0.0001. (F) Patterns of nuclear positioning. Data shown in (E) are presented as columns. The sample numbers are shown on the bottom of each column. p values were obtained from the two-tailed χ^2^ test. ∗∗∗∗p < 0.0001.

We wished to identify the mechanism by which nuclear displacement is elicited. As the nucleus moves in accordance with CAR assembly, we treated *cut7* cells with an F-actin depolymerizing drug, latrunculin A (LatA, 50 μM) at 36°C. Remarkably, LatA-treated *cut7* mutant cells almost completely ceased nuclear displacement (Figure 3A; see Video S5). Figure 3B shows the kinetics of nuclear movement in the absence or presence of LatA. In all cases examined (n = 12), LatA treatment displayed very minimal nuclear fluctuations (Figure 3C). By contrast, in the absence of LatA (n = 18), ∼44% or ∼11% cells displayed the maximal distance of >1 μm or 0.5–1.0 μm, respectively (Figure 3C). We also treated *nda3-1828*, *nuc2-663,* and *cut9-665* cells with LatA at 36°C and found that as in the case for *cut7*, nuclear displacement was suppressed in these cells by actin depolymerization (Figures S3A and S3B). These results indicated that the F-actin cytoskeleton is responsible for mitotic nuclear displacement and suggested that the driving force is generated by the polymerized actin structures.

Video S5. Suppression of the nuclear displacement in cut7-22 by treatment with LatA, related to figure 3

In fission yeast, F-actin assembles into three different structures during vegetative growth cycles: filamentous actin cables, endocytic actin patches and the CAR (Kovar et al., 2011) (see Figure 2A). To distinguish which actin structures are responsible for force generation, we treated *cut7-22* cells with CK-666, a small molecule that specifically inhibits the Arp2/3 complex, thereby disassembling actin patches (Burke et al., 2014; Nolen et al., 2009). Addition of this inhibitor resulted in the loss of actin patches as expected (Figure 3D). Nonetheless, nuclear displacement still occurred (Figures 3E and 3F). Notably, the degree of nuclear displacement was augmented by CK-666 treatment. This indicates that endocytic actin patches are not essential for force generation and in fact they play a suppressing role in nuclear movement.

### Cdc12/Formin, Myo2, and Myo51 are responsible for nuclear displacement

**Figure 4:**
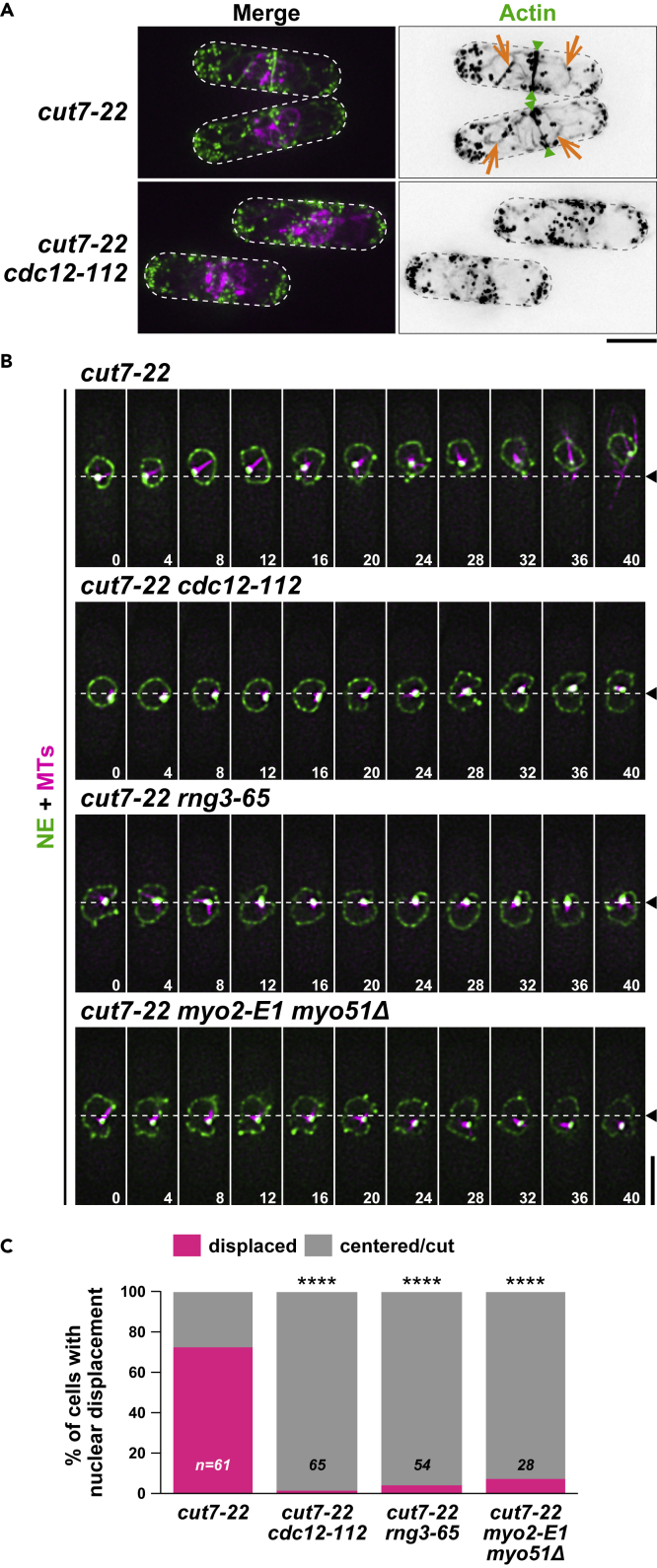
Cdc12/Formin, Myo2, and Myo51 are responsible for nuclear displacement (A) Lack of actin cables and the CAR in *cut7-22cdc12-112* cells. *cut7-22* or *cut7-22cdc12-112* cells containing mCherry-Atb2 (MTs, magenta), Cut11-mRFP (the NE, magenta), and LifeAct-GFP (actin, green) were grown at 27°C and shifted to 36°C for 3 h. Actin cables (orange arrows) and the CAR (green arrowheads) are pointed. (B) Time-lapse images of the nuclear position. Indicated mutant cells grown at 27°C were incubated at 36°C for 2 h, when live imaging started. Individual mutants contain mCherry-Atb2 (magenta, MTs) and Cut11-GFP (green, NE). The numbers on the bottom right indicate times in minutes upon recording. The position of the cell center is shown with dotted lines and arrowheads. Scale bars, 5 μm (A and B). See also Videos S6, S7, and S8. (C) Profiles of the relative position of the nucleus in individual mutants. If x (the distance between the center of the cell axis and that of the nucleus) was <1 μm or a cell showed cut, it was classified as centered/cut. If x was >1 μm, it was classified as displaced. The sample numbers are shown on the bottom of each column. All p values were obtained from the two-tailed χ^2^ test. ∗∗∗∗p < 0.0001. See also Figure S4.

Next, we used the *cdc12-112* ts mutant; *cdc12* encodes Formin that is required for assembly of mitotic actin cables and the CAR (Chang et al., 1997; Huang et al., 2012). We constructed *cut7-22cdc12-112* double mutants, which were indeed defective in actin cable assembly and CAR formation (Figure 4A). In this background, nuclear movement was almost completely suppressed (Figures 4B and 4C; see Video S6), which recapitulated the situation of LatA treatment shown earlier (see Figure 3A).

Video S6. Suppression of nuclear displacement in cut722cdc12-112, related to figure 4

We then addressed the roles of myosin. Using the *rng3-65* ts mutant (Lord and Pollard, 2004; Wong et al., 2000), which is defective in myosin assembly and activation, we found that the nucleus remained in the middle of *cut7-22rng3-65* mutant cells (Figures 4B and 4C; see Video S7). Furthermore, a combination of mutations in myosins II and V (*myo2-E1* and *myo51* deletion) suppressed nuclear displacement in *cut7-22* (Figures 4B and 4C; see Video S8). This result is in line with the previous data showing that Myo2 and Myo51 together collaborate to form proper mitotic actin cables and the CAR (Huang et al., 2012). Myo1 and Myo52 (another myosin V) appear unimportant, as *cut7-22myo1Δ*, *cut7-22myo52Δ,* or *cut7-22myo2-E1myo52Δ* cells exhibited nuclear displacement (Figures S4A and S4B). In clear contrast to the requirement of Cdc12, another Formin For3, which promotes actin cable formation mainly during interphase (Feierbach and Chang, 2001), is dispensable for nuclear movement (Figures S4A and S4B). These results show that mitotic Cdc12/Formin that assembles cables and the CAR and two types of myosins (Myo2 and Myo51) are responsible for nuclear displacement.

Video S7. Suppression of nuclear displacement in cut7-22rng3-65, related to figure 4

Video S8. Nuclear displacement in the cut7-22myo2-E1myo51Δ mutant, related to figure 4

### Mitotic actin cables, but not actomyosin ring constriction, drive nuclear displacement

**Figure 5:**
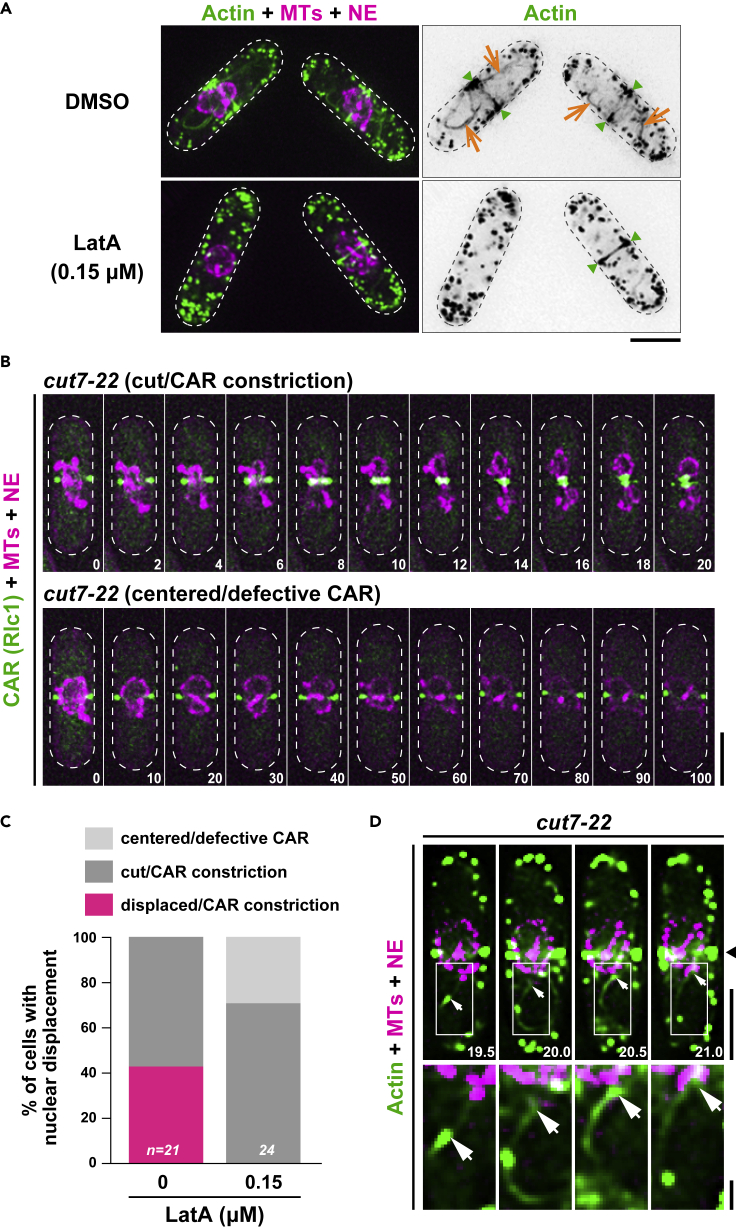
Mitotic actin cables, but not actomyosin ring constriction, drive nuclear displacement (A) Selective disappearance of actin cables by treatment with a low concentration of LatA. *cut7-22* cells containing mCherry-Atb2 (MTs, magenta), Cut11-mRFP (the NE, magenta), and LifeAct-GFP (actin, green) were grown at 27°C and shifted to 36°C for 2 h when LatA (0.15 μM) or DMSO was added. Still images were taken 10 min after LatA or DMSO addition. Actin cables (orange arrows) and the CAR (green arrowheads) are pointed. (B) Time-lapse images of the nuclear position in *cut7-22* cells treated with a low concentration of LatA. *cut7-22* cells containing mCherry-Atb2 (MTs, magenta), Cut11-mRFP (the NE, magenta), and Rlc1-GFP (the CAR, green) incubated as in (A) were imaged. Representative cells that successfully underwent CAR constriction (top) or failed to assemble/constrict the CAR (bottom) are shown. The numbers on the bottom right indicate times in minutes upon recording. Scale bars, 5 μm (A and B). (C) Suppression of nuclear displacement under a low concentration of LatA. If x (the distance between the center of the cell axis and that of the nucleus) was <1 μm or a cell showed cut, it was classified as centered/cut. If x was >1 μm, it was classified as displaced. The sample numbers are shown on the bottom of each column. (D) Visualization of interaction between F-actin cables and the nuclear envelope in *cut7-22*. A *cut7-22* strain used in (A) was grown at 27°C and incubated at 36°C for 2 h, when time-lapse imaging started (see Video S9). The bottom row shows enlarged images in squares shown on the top row. The numbers on the bottom right indicate times in minutes upon recording. Arrows point the tips of F-actin cables (green) that interact with the NE (magenta). The position of the cell center is shown with an arrowhead. Scale bars, 5 μm (top) and 1 μm (bottom).

To distinguish the requirement of actin cables and the CAR for nuclear movement, we treated *cut7-22* cells with a lower concentration (0.15 μM) of LatA, the condition of which reportedly blocks actin cable formation, whereas CAR assembly and constriction proceed (Gomez-Gil et al., 2020; Tournier et al., 2004). We found that under this low concentration of LatA, mitotic actin cables disappeared and ∼70% of *cut7-22* cells (17/24) were capable of assembling and constricting the CAR (Figures 5A–5C). Intriguingly, all cells undergoing CAR constriction as well as those defective in CAR assembly/constriction failed to displace the nucleus (Figures 5B and 5C). This result supports the notion that mitotic actin cables, but not CAR constriction, drive the initial nuclear displacement.

Close inspection of time-lapse movies during short intervals captured time points at which actin cables interacted with the NE in the *cut7-22* mutant (Figure 5D; see Video S9). Although it is possible that the nucleus is displaced poleward through myosin-mediated pulling force (Lo Presti et al., 2012), as interaction between actin cables and the NE appears transient, we interpret this result as a hint that mitosis-specific F-actin cables push the nucleus, leading to nuclear displacement upon prolonged mitotic arrest.

Video S9. A cut7-22 cell in which F-actin cables interact with the nuclear envelope, related to figure 5

### Inhibition of actomyosin ring constriction substantially but not completely abolishes nuclear displacement

**Figure 6:**
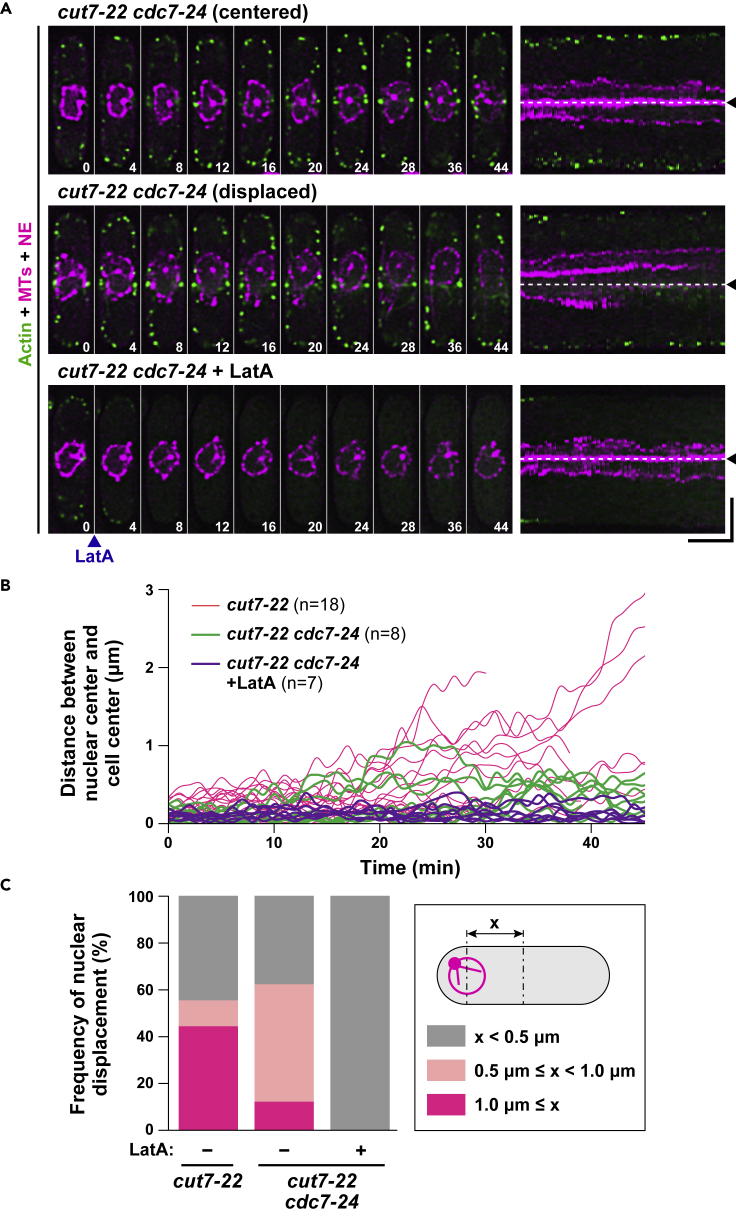
Inhibition of actomyosin ring constriction substantially but not completely abolishes nuclear displacement (A) Time-lapse and kymograph images of the nuclear position in *cut7-22cdc7-24* cells incubated at 36°C in the absence or presence of LatA (see Videos S10 and S11). Mutant cells were grown at 27°C, shifted to 36°C, and incubated for 2 h, when imaging started. Two representative cells, in which the nucleus remains centered (top row) or is displaced (middle row), are shown. LatA (50 μM) was added at time 0. Cells contain mCherry-Atb2 (magenta, MTs), Cut11-mRFP (magenta, the NE), and LifeAct-GFP (green, actin). Corresponding kymograph images are shown on the right, in which the middle of the cell axis is shown with dotted lines and arrowheads. The numbers on the bottom right indicate times in minutes upon recording. Scale bars, 10 min (horizontal) and 5 μm (vertical). (B) Profiles of the relative position of the nucleus in *cut7-22cdc7-24* mutants in the absence (n = 8) or presence (n = 7) of LatA. The distance (x) between the center of the cell axis and that of the nucleus was measured at each time point and plotted against time. (C) Classification of the patterns of nuclear movement in *cut7-22cdc7-24* cells in the absence or presence of LatA. The percentage of cells that do or do not show nuclear displacement is shown. For each sample, the maximal distance (x μm) between the center of the cell axis and that of the nucleus was determined using the data shown in (B) and categorized into three classes: displaced (shown in magenta, in which x is ≥ 1 μm), mildly displaced (shown in pink, in which x is between 0.5 μm and 1 μm), and centered (shown in gray, in which x is ≤ 0.5 μm). The data for *cut7-22* in (B) and (C) are the same as those in Figures 3B and 3C, respectively.

Although the nucleus initiates displacement coincident with CAR assembly before its constriction (see Figure 2C), the process of ring closure might cooperate to promote nuclear movement. To scrutinize this possibility, we constructed double mutants between ts *cut7-22* and *cdc7-24* (Nurse et al., 1976). The Cdc7 kinase is a component of the SIN (Septation Initiation Network) that is required for not only septum formation but also maturation and constriction of the CAR (Krapp and Simanis, 2008; Roberts-Galbraith and Gould, 2008). Upon incubation of *cut7-22cdc7-24* mutant cells at 36°C, nuclear movement was imaged with time-lapse microscopy. As shown in Figures 6A and 6B, the degree of nuclear movement was substantially suppressed. Nonetheless, we noticed residual movement (Figure 6C; see Video S10). Next, we added LatA to *cut7-22cdc7-24* mutant cells upon shift to 36°C. As expected, LatA almost completely halted nuclear movement (Figures 6A–6C; see Video S11).

Video S10. Nuclear displacement in cut7-22cdc7-24, related to figure 6

Video S11. Suppression of the nuclear displacement in cut7-22cdc7-24 by treatment with LatA, related to figure 6

We asked whether SAC activation is necessary for nuclear movement in *cut7-22cdc7-24* cells. Interestingly, albeit modest, significant nuclear displacement still occurred in *cut7-22cdc7-24mad2Δ* cells (Figure S2). Thus, the nucleus is capable of being displaced from the cell center even in the absence of mitotic delay and CAR constriction as long as cells encounter mitotic blockage. Taken together, we posit that nuclear displacement takes place initially by a force generated through F-actin cables that elongate toward the medial nuclear region as they incorporate into the CAR and then the movement is further accelerated by a force derived from CAR constriction.

### *cut7* survivors become diploidized

**Figure 7:**
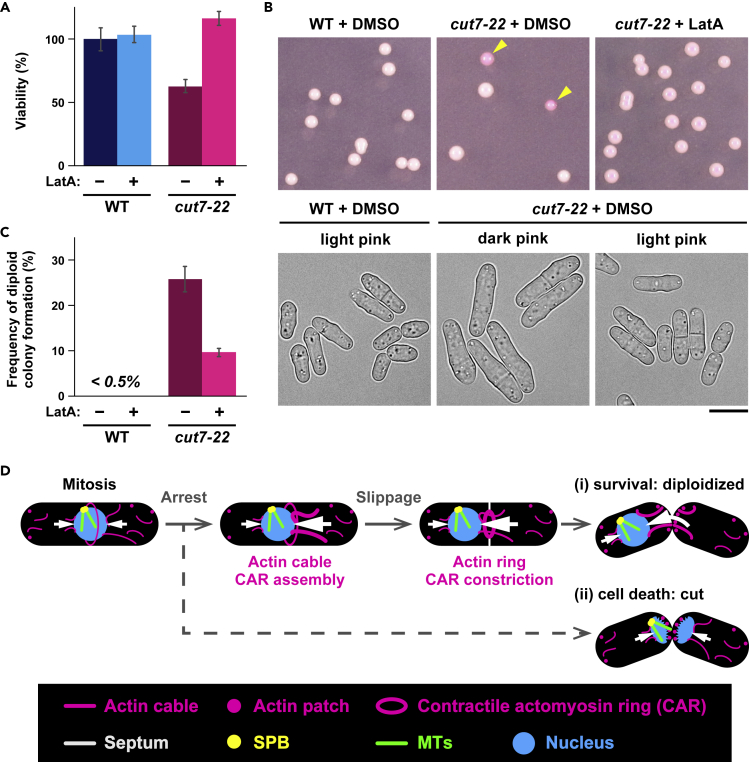
*cut7* survivors become diploidized (A) Viability of wild-type and *cut7-22* cells incubated at the restrictive temperature in the absence or presence of LatA. Wild-type and *cut7-22* cells grown at 27°C were shifted to 36°C. After 2-h incubation at 36°C, DMSO or LatA (50 μM) was added, and cultures were incubated for additional 1 h. The cell number was measured, and after appropriate dilutions, cells were spread on rich YE5S plates containing Phloxine B. Plates were then incubated at 27°C to assess colony-forming ability (viability: the number of colonies formed divided by that of cells spread, and the value of wild-type cells were set as 100%). Data are presented as the means ± SEM (≥100 colonies). (B) Representative pictures of plates that contain survivor colonies (wild-type, left; *cut7-22* cells, middle and right) are shown. Dark pink colonies (diploids) are pointed with arrowheads. Cell morphologies derived from light and dark pink colonies are shown on the bottom. Scale bar, 10 μm. (C) The percentage of dark pink colonies (diploid). At least 30 colonies were counted in three independent experiments. Data are presented as the means ± SEM. (D) A model depicting controlling mechanisms of mitotic nuclear positioning. During mitosis, actin cables nucleate in the cytoplasm and are recruited toward the cell center to be incorporated into the CAR. This process may generate a pushing force (white arrows) toward the nucleus. When mitosis is blocked, forces from either side of the nucleus become imbalanced, leading to nuclear displacement. Upon prolonged delay, cells are destined for two fates. In one type (i), the nucleus is further displaced from the cell center imposed by CAR constriction. Cells with displaced nuclei give rise to diploid and anucleate progenies. In another type (ii), the nucleus stays in the middle, leading to the lethal cut phenotype.

We next asked whether the inhibition of CAR assembly/cytokinesis could rescue cut-mediated lethality of *cut7-22* cells. To this end, *cut7-22* mutants were cultured at 36°C in the absence or presence of LatA (50 μM). Note that LatA not only suppresses nuclear displacement but also inhibits CAR assembly. Remarkably, in *cut7-22* mutant cells, LatA substantially increased viability (Figure 7A). Thus, inhibition of CAR assembly and cytokinesis largely rescued lethality derived from the cut phenotype.

In the course of scoring the number of colonies formed at 27°C, we realized that many of *cut7-22* survivor colonies stained dark pink on Phloxine B-containing plates (Figures 7B and 7C), implying that they are diploid (Phloxine B stains diploid colonies with dark pink colors). In fact, visual inspection of cell morphology of these *cut7-22* survivors showed that these cells were wider and longer, reminiscent of diploid fission yeast cells (Figure 7B). Intriguingly, *cut7* colonies that were formed from cultures incubated in the presence of LatA were mostly haploids (Figures 7B and 7C). These results indicate that first, the main reason for lethality of *cut7* is derived from the cut phenotype; second, some cells could escape from cut by displacing the nucleus from the middle of the cell axis; and finally, these *cut7* survivors could resume cell division as diploid progenies at the permissive temperature. Hence, nuclear displacement provides an advantage to mitotically arrested cells for survival; however, it is brought about in exchange for genome instability.

## Discussion

In this study, we have shown that the nucleus becomes displaced in various fission yeast mitotic mutants. It is known that nuclear positioning during interphase in fission yeast is driven by the cytoplasmic MTs (Daga et al., 2006b; Tran et al., 2001); hence, the MT would be a prime suspect for mitotic nuclear positioning. However, MT morphologies and dynamics are totally different among individual mitotic mutants. Therefore, a force responsible for nuclear displacement upon mitotic arrest is unlikely to stem from the MT cytoskeleton. Subsequent experiments have unequivocally established that the actin cytoskeleton instead drives mitotic nuclear movement. More precisely, mitotic actin cables and CAR constriction are responsible; Formin mutants (*cdc12*) with failure in assembling mitotic F-actin cables and the CAR (Huang et al., 2012) suppressed nuclear movement. Consistent with this result, live imaging indicates that upon mitotic arrest, F-actin cables nucleating from the cytoplasm migrate toward the cell center and appear to interact with the NE. This physical contact may generate a pushing force toward the nucleus. We ponder that under normal conditions, the tips of F-actin cables push both sides of the NE randomly, retaining the nucleus in the cell center during the short period of mitosis. Upon prolonged mitotic arrest, however, pushing forces elicited by mitotic actin cables become imbalanced, leading to nuclear displacement (Figure 7D), although currently the mechanism underlying the generation of force imbalance remains elusive.

A force generated through CAR constriction also plays an important role in nuclear displacement. However, ring closure does not act as a trigger. Instead, it accelerates the movement; only the nucleus that is already off-center further slips away as a reaction to the pushing force derived from CAR constriction. Recently, a novel mitotic actin structure is reported, in which it is formed around the nucleus as the CAR matures during anaphase (Schutt and Moseley, 2020). This structure might collaborate with actin cables and/or the CAR, thereby displacing the nucleus. Overall, mitotic F-actin plays a dual role; one is required for successful cell division and the other involves the protection from lethal mitotic catastrophe upon mitotic arrest and exit. It, however, remains possible that the second role stems from a by-product of normal actin rearrangements: incorporation of centripetal mitotic actin cables into the actomyosin ring and CAR constriction.

The SAC inhibits sister chromatid separation and mitotic exit through APC/C inactivation (Musacchio, 2015). Although the nucleus is capable of being displaced in the absence of SAC function, F-actin-mediated nuclear displacement appears to help evade a catastrophic consequence when activated SAC is silenced and/or the septation occurred without chromosome segregation. However, this salvage is inefficient and a double-edged sword, as the nucleate compartment would resume cell division as diploids and the other anucleate part dies (Figure 7D). In addition, some cells that escape from cut unsuccessfully would become aneuploids, nearly lethal in fission yeast and cancer-prone in humans (Niwa et al., 2006; Orr et al., 2015).

Previous work in fission yeast suggested that F-actin impacts spindle elongation rate and the fidelity of sister chromatid segregation (Gachet et al., 2004; Tournier et al., 2004). A favored model in which the CAR physically interacts with the astral MTs during mid-mitosis came to question and is under reconsideration (Zimmerman et al., 2004); therefore, a mechanism by which polymerized actin regulates mitotic progression remains unsolved. Given our findings, one possible explanation for these previous observations would be that the dynamic inward movement of F-actin produces a pushing force toward the nucleus, thereby ensuring proper spindle orientation and MT-kinetochore attachment.

A wide range of organisms position the mitotic nucleus/chromosomes to defined locations within cells. In budding yeast mitosis, the nucleus translocates from the mother cell compartment to the bud neck, thereby positioning the mitotic spindle along the mother-daughter axis. This dynamic process requires interaction between F-actin cables and astral MTs (Caydasi et al., 2010; Fraschini et al., 2008). In vertebrate oocytes, actin diffusion centers the nucleus during prophase I and meiosis I (Almonacid et al., 2015, 2019). In human cell cultures, the actin cytoskeleton collaborates with the dynein-based machinery that exerts cortical pulling forces on astral MTs, thereby positioning the nucleus at the center of the cell (Kiyomitsu, 2019). In the moss *Physcomitrella patens*, nuclear positioning is regulated by collaborative actions between MTs and F-actin (Yi and Goshima, 2020). Whether F-actin cables directly drive movement of nucleus/chromosomes in other systems is of great interest to explore. Collectively, F-actin and its dynamics play more multifaceted roles in the maintenance of genome integrity than is currently thought.

### Limitations of the study

Potential caveats of the work may include the following points. (1) Nuclear displacement may not be an active cellular strategy; instead, it could be a by-product of normal actin rearrangements. (2) The mechanism by which the nucleus is displaced has not fully been uncovered. (3) The phenomenon observed in this study could be restricted to fission yeast.

### Resource availability

#### Lead contact

Further information and requests for resources and reagents should be directed to and will be fulfilled by the Lead Contact, Takashi Toda (takashi-toda@hiroshima-u.ac.jp).

#### Materials availability

This study did not generate new unique reagents.

#### Data and code availabilty

All images can be found online at https://data.mendeley.com/drafts/htsvnm6hws (https://doi.org/10.17632/htsvnm6hws.1).

## Methods

All methods can be found in the accompanying Transparent Methods supplemental file.

## Supplemental information

Document S1. Transparent methods, figures S1–S4, and table S1
