# Supplementary material for: Escape from mitotic catastrophe by actin-dependent nuclear displacement in fission yeast
Source: iScience. 2021 Jan 1;24(1):102031. doi: 10.1016/j.isci.2020.102031 (PMC7814194; doi:10.1016/j.isci.2020.102031)
Supplement: Document S1. Transparent methods, figures S1–S4, and table S1 [file mmc1.pdf]

iScience, Volume *24*

Supplemental Information

Escape from mitotic catastrophe
by actin-dependent nuclear displacement
in fission yeast

Masashi Yukawa, Yasuhiro Teratani, and Takashi Toda

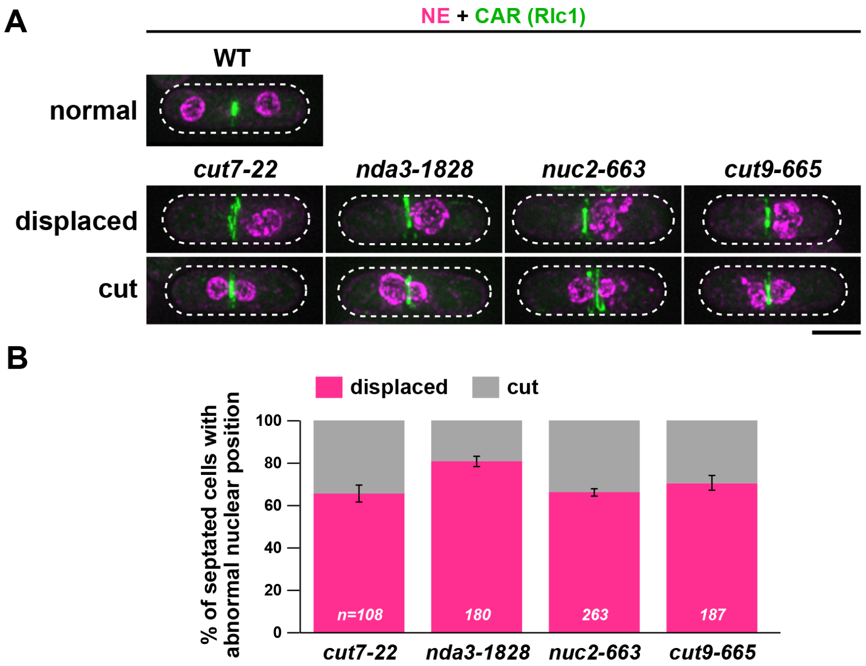

Figure S1. Nuclear displacement in several mitotic mutant cells (Related to Figure 1).

(A) The position of the nucleus. Indicated strains contain Cut11-RFP (a component of the nuclear envelope, NE) and Rlc1-GFP (Type II myosin regulatory light chain and a marker for the CAR). Exponentially growing wild type or mitotic mutants grown at 27° C were shifted to 36° C and incubated for 3 h except for *nda3-1828*, which was incubated for 6 h. Representative cells for wild type (top row), mutants displaying displaced nuclei (middle row) or cut (bottom row) are shown. Scale bar, 5 µm. (B) The percentage of cells displaying nuclear displacement or cut. The sample numbers (n) for individual strains are indicated on the bottom of columns. Data are presented as the means ± S.D.

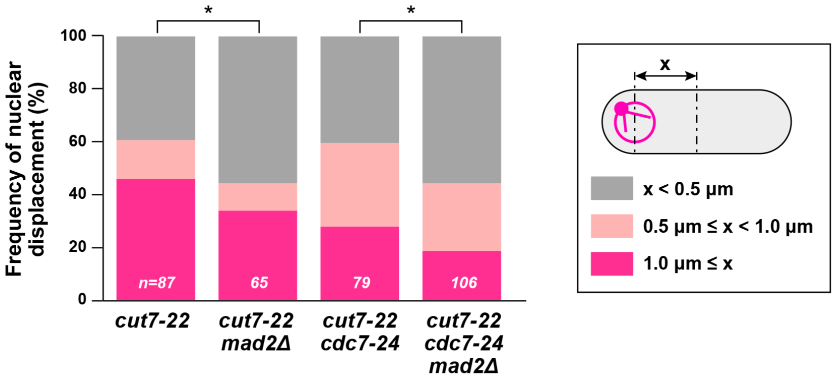

Figure S2. Impact of the spindle assembly checkpoint on mitotic nuclear movement (Related to Figure 2).
The degree of nuclear movement in *cut7-22* or *cut7-22cdc7-24* mutants in the presence or absence of *mad2*. Individual strains grown at 27 ° C were shifted to 36 ° C. After 3 h incubation, the position of the nucleus was determined. The percentage of cells that do or do not show nuclear displacement is shown. For each sample, the distance (x µm) between the center of the cell axis and that of the nucleus was determined and categorized into three classes: displaced (shown in magenta, in which x is ≥1 µm), mildly displaced (shown in pink, in which x is between 0.5 µm and 1 µm) and centered (shown in gray, in which x is ≤0.5 µm). The sample number is shown on the bottom of each column. *p*-values were obtained from the two-tailed χ^2 test: *p <0.05.

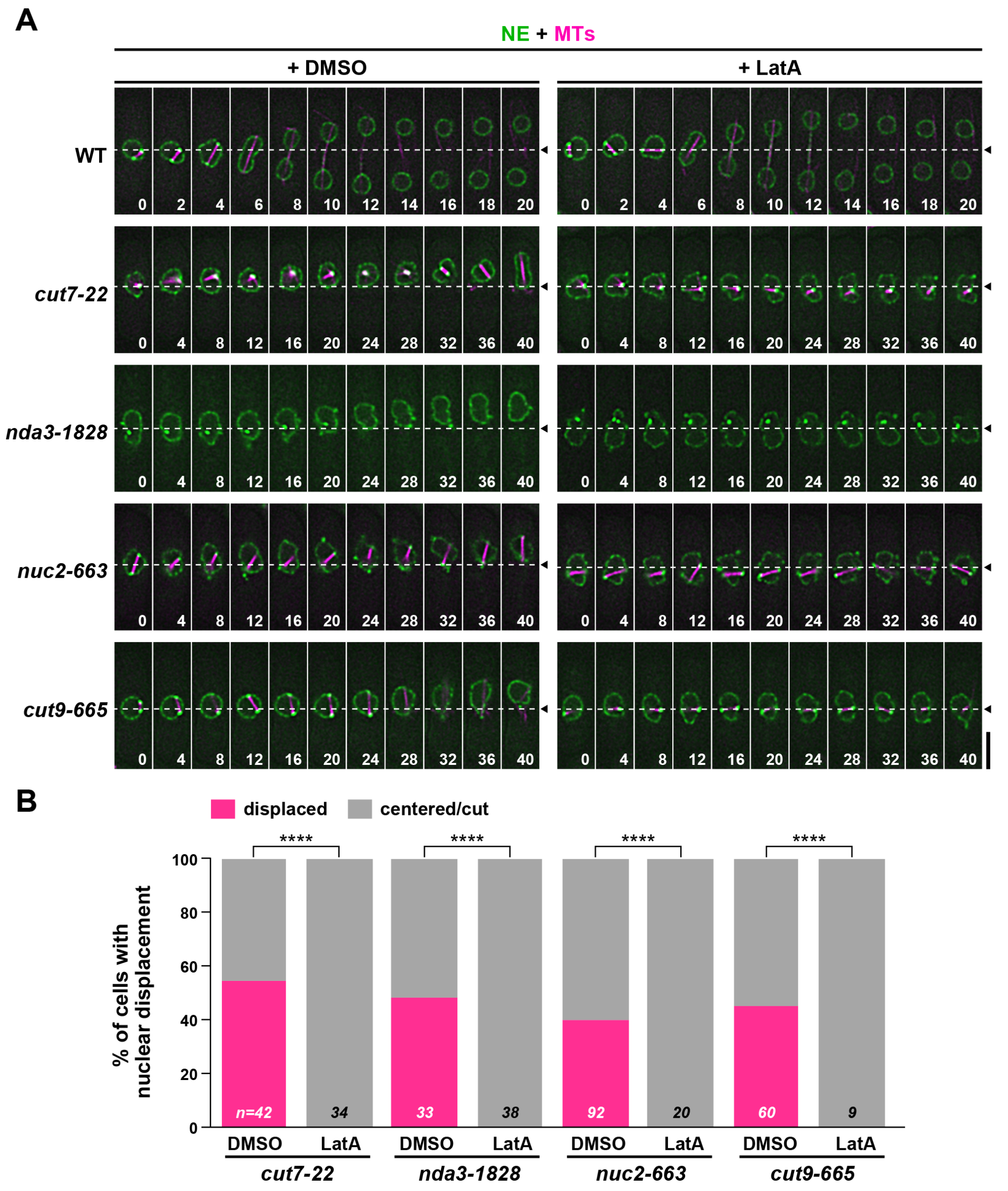

Figure S3. Suppression of nuclear displacement in various mitotic mutants with depolymerization of the F-actin cytoskeleton (Related to Figure 3).
(A) Time-lapse images of the nuclear position in indicated mutant cells in the absence or presence of LatA. Wild-type and individual mutant cells grown at 27°C were shifted to 36°C and incubated for 2 h except for *nda3-1828*, which was incubated for 5 h. DMSO or LatA (50 µM) was then added and cells were incubated for further 10 min, when imaging started (time 0). The numbers on the bottom right corners of each image show times in minutes. Cells contained mCherry-Atb2 (magenta, MTs) and Cut11-GFP (green, the NE). The middle of the cell axis is shown with dotted lines and arrowheads. Scale bar, 5 µm. (B) Profiles of nuclear positioning in individual mitotic mutants in the absence or presence of LatA. For each strain, the maximal distance (x µm) between the center of the cell axis and that of the nucleus at each time point was determined. If x was kept <1 µm for longer than 20 min or a cell showed cut, it was classified as centered/cut. If x was >1 µm during observation, it was classified as displaced. The sample numbers (n) are shown on the bottom for each column. All *p*-values were obtained from the two-tailed χ^2 test: **** p <0.0001.

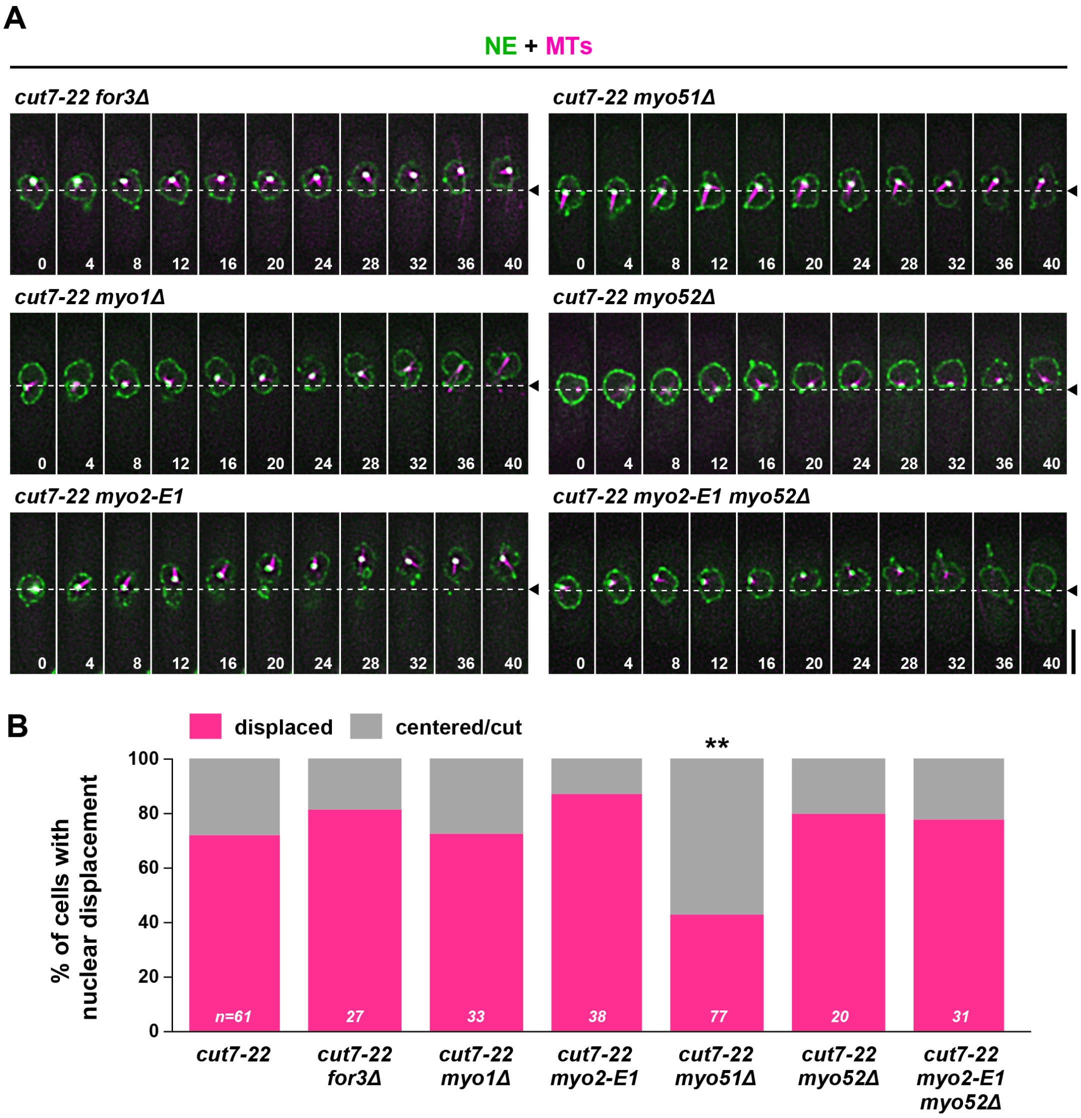

Figure S4. Myo51 is important for nuclear positioning, but For3/Formin or Myo52 is not involved (Related to Figure 4).

(A) Time-lapse images of the nuclear position. Indicated mutant cells grown at 27 ° C were incubated at 36 ° C for 2 h, when live imaging started. Individual mutants contain mCherry-Atb2 (MTs, magenta) and Cut11-GFP (NE, green). The position of the cell center is shown with dotted lines and arrowheads. Scale bar, 5 µm. (B) Profiles of the relative position of the nucleus. If the distance (x µm) between the center of the cell axis and that of the nucleus was <1 µm or a cell showed cut, it was classified as centered/cut (gray). If x was >1 µm, it was classified as displaced (magenta). The sample numbers are shown on the bottom of each column. *p*-values were obtained from the two-tailed χ^2 test: ** $p < 0.01$.

LEGENDS FOR SUPPLEMENTAL VIDEOS

Supplementary Video S1: Related to Figure 2

Supplementary Video S2: Related to Figure 2

Supplementary Video S3: Related to Figure 2

Supplementary Video S4: Related to Figure 2

Supplementary Video S5: Related to Figure 3

Supplementary Video S6: Related to Figure 4

Supplementary Video S7: Related to Figure 4

Supplementary Video S8: Related to Figure 4

Supplementary Video S9: Related to Figure 5

Supplementary Video S10: Related to Figure 6

Supplementary Video S11: Related to Figure 6

Supplemental Table S1: Fission yeast strains used in this study (Related to Figures 1-7, S1-4, and Videos S1-11).

Strains	Genotypes	Figures used	Derivations
513	*h⁻ leu1 ura4*	1A	Our lab stock
YY146	*h⁺ cut7-22 leu1 ura4 his2*	1A-B	Our lab stock
MY2300	*h⁻ nda3-1828 leu1 ura4*	1A-B	Our lab stock
MS072	*h⁻ nuc2-663 alp7-GFP-kanR mis6-3HA-kanR leu1 ura4*	1A-B	Our lab stock
KSH544	*h⁻ cut9-665 leu1 ura4 his2*	1A-B	Our lab stock
MY1008	*h⁻ cut7-21 leu1 ura4*	1B	Our lab stock
YT098	*h⁻ cut7-123-GFP-kanR leu1 ura4*	1B	Our lab stock
MY1691	*h⁻ cut7-446 leu1 ura4*	1B	Our lab stock
YT656	*h⁺ cut11-4mRFP-natR aur1::aur1R-Pnda3-mCherry-atb2 Pact1-lifeact-GFP::leu1⁺ leu1 ura4 ade6*	2B, 7A-C	This study
YT657	*h⁺ cut7-22 cut11-4mRFP-natR aur1::aur1R-Pnda3-mCherry-atb2 Pact1-lifeact-GFP::leu1⁺ leu1 ura4 ade6? his2*	2B, 3A-F, 4A, 5A, 5D, 6B-C, 7A-C	This study
MY2313	*h⁻ cut7-22 cut11-4mRFP-natR aur1::aur1R-Pnda3-mCherry-atb2 rlc1-GFP-ura4⁺ leu1 ura4*	2C, 5B-C, S2	This study
MY2429	*h⁻ cut7-22-GFP-kanR cdc12-112 cut11-4mRFP-natR aur1::aur1R-Pnda3-mCherry-atb2 pAct1-Lifeact-GFP::leu1⁺ leu1 ura4 ade6?*	4A	This study
MY2397	*h⁻ cut7-22-GFP-kanR cut11-GFP-ura4⁺ aur1::aur1R-Pnda3-mCherry-atb2 leu1 ura4 ade6*	4B-C, S4A	This study
MY2394	*h⁻ cut7-22-GFP-kanR cdc12-112 cut11-GFP-ura4⁺ aur1::aur1R-Pnda3-mCherry-atb2 leu1 ura4*	4B-C	This study
MY2393	*h⁻ cut7-22-GFP-kanR rng3-65 cut11-GFP-ura4⁺ aur1::aur1R-Pnda3-mCherry-atb2 leu1 ura4 ade6?*	4B-C	This study
MY2540	*h⁻ cut7-22 myo2-E1 myo51::kanR cut11-GFP-ura4⁺ aur1::aur1R-Pnda3-mCherry-atb2 leu1 ura4 ade6?*	4B-C	This study
YT703	*h⁺ cut7-22 cdc7-24 cut11-4mRFP-natR aur1::aur1R-Pnda3-mCherry-atb2 Pact1-lifeact-GFP::leu1⁺ leu1 ura4 his2*	6A-C, S2	This study
MY2309	*h⁻ cut11-4mRFP-natR rlc1-GFP-ura4⁺ leu1 ura4*	S1A	This study
MY2311	*h? cut7-22 cut11-4mRFP-natR rlc1-GFP-ura4⁺ leu1 ura4*	S1A-B	This study
MY2373	*h⁻ nda3-1828 cut11-4mRFP-natR rlc1-GFP-ura4⁺ leu1 ura4*	S1A-B	This study
MY2329	*h? nuc2-663 cut11-4mRFP-natR rlc1-GFP-ura4⁺ mis6-3HA-kanR leu1 ura4*	S1A-B	This study
MY2325	*h⁻ cut9-665 cut11-4mRFP-natR rlc1-GFP-ura4⁺ leu1 ura4*	S1A-B	This study
MY2500	*h⁻ cut7-22 mad2::LEU2 cut11-4mRFP-natR aur1::aur1R-Pnda3-mCherry-atb2 leu1 ura4*	S2	This study
MY2535	*h⁺ cut7-22 cdc7-24 mad2::LEU2 cut11-4mRFP-natR aur1::aur1R-Pnda3-mCherry-atb2 leu1 ura4 his2*	S2	This study
MO209	*h⁻ cut11-GFP-ura4⁺ aur1::aur1R-Pnda3-mCherry-atb2 leu1 ura4*	S3A	Our lab stock

YT661	h^- *cut7-22 cut11-GFP-ura4*$^+$ *aur1::aur1R-Pnda3-mCherry-atb2 leu1 ura4*	S3A-B	This study
MY2369	h^- *nda3-1828 cut11-GFP-ura4*$^+$ *aur1::aur1R-Pnda3-mCherry-atb2 leu1 ura4*	S3A-B	This study
MY2361	$h^?$ *nuc2-663 cut11-GFP-ura4*$^+$ *aur1::aur1R-Pnda3-mCherry-atb2 leu1 ura4*	S3A-B	This study
MY2365	h^- *cut9-665 cut11-GFP-ura4*$^+$ *aur1::aur1R-Pnda3-mCherry-atb2 leu1 ura4*	S3A-B	This study
MY2399	h^+ *cut7-22-GFP-kanR for3::ura4*$^+$ *cut11-GFP-ura4*$^+$ *aur1::aur1R-Pnda3-mCherry-atb2 leu1 ura4 ade6? his2*	S4A-B	This study
MY2483	h^- *cut7-22 myo1::kanR cut11-GFP-ura4*$^+$ *aur1::aur1R-Pnda3-mCherry-atb2 leu1 ura4 ade6?*	S4A-B	This study
MY2404	h^- *cut7-22-GFP-kanR myo2-E1 cut11-GFP-ura4*$^+$ *aur1::aur1R-Pnda3-mCherry-atb2 leu1 ura4 ade6?*	S4A-B	This study
MY2481	h^- *cut7-22 myo51::kanR cut11-GFP-ura4*$^+$ *aur1::aur1R-Pnda3-mCherry-atb2 leu1 ura4 ade6?*	S4A-B	This study
MY2547	h^- *cut7-22 myo52::kanR cut11-GFP-ura4*$^+$ *aur1::aur1R-Pnda3-mCherry-atb2 leu1 ura4*	S4A-B	This study
MY2542	h^- *cut7-22 myo2-E1 myo52::kanR cut11-GFP-ura4*$^+$ *aur1::aur1R-Pnda3-mCherry-atb2 leu1 ura4 ade6?*	S4A-B	This study

*Strains were developed for this study unless otherwise specified.

his2=his2-245; *leu1=leu1-32*; *ura4=ura4-D18*; *ade6=ade6-M210* or *ade6-M216*.

TRANSPARENT METHODS

Strains, media and genetic methods

Fission yeast strains used in this study are listed in Table S1. Cells were grown under standard conditions as previously described (Moreno et al., 1991; Sato et al., 2005). For all experiments, rich YE5S plates and media were used. Viability assay was performed by spreading 100 cells on YE5S plates containing Phloxine B and incubated at 27˚C for 3 d. Viability was calculated by counting the number of colonies formed on plates. Colonies displaying dark red colours were judged as diploids, which was confirmed by observations of cell morphologies with microscopic inspection.

Depolymerization of the actin cytoskeleton

Stock solutions of 10 mM Lat A (Latruncurin A, FUJIFILM Wako Pure Chemical Corporation, Japan, 125-04363) and 20 mM CK-666 (Sigma-Aldrich, St. Louis, MO, U. S. A., SML0006) were prepared in DMSO. To depolymerize F-actin filaments, Lat A (0.15 or 50 μM) was added to cells incubated at 36˚C for 2 h and cultures were kept at this temperature. To disassemble the actin patches, cells were pre-incubated at 36°C

for 2 h, followed by an additional 20 min with 100 μM CK-666. For mock treatment, the same amount of DMSO was added as a control.

Fluorescence microscopy and sample preparation

Fluorescence microscopy images were acquired using one of two DeltaVision wide-field inverted epifluorescence systems (GE Healthcare, Chicago, IL, U. S. A.). Cells were imaged using an Olympus IX71 microscope comprised of an Olympus Plan Apo 60x, NA 1.42 oil immersion objective and a CoolSNAP HQ2 charge-coupled device camera (Photometrics, Tucson, AZ, U. S. A.). All image acquisition and subsequent deconvolution were performed using SoftWorx 6.5.2 software (GE Healthcare, Chicago, IL, U. S. A.). Images were analyzed in SoftWorx and processed with Adobe Photoshop CS6 and Adobe Illustrator CS6. For live cell time-lapse imaging, log-phase cultures grown at 27˚C were shifted to 36˚C for 2 h prior to imaging at 36˚C. Live cells were adhered to a glass-bottomed culture dish (MatTek Corporation, Ashland, MA, U. S. A.) using soybean lectin and covered with warmed YE5S media. Fixed cells were imaged on glass slides. Cells were fixed using cold methanol and acetone and resuspended in PBS, followed by staining for DNA with 4',6-diamidine-2'-phenylindole (DAPI, Sigma-Aldrich, St. Louis, MO, U. S. A., D9542) and Calcofluor White (American Cyanamid Co., Princeton, NJ, U. S. A., M2R). Kymographs and movies were generated using Adobe Photoshop CS6.

Quantification and Statistical analysis

We measured the distance between the center of the cell axis and that of the nucleus by using SoftWorx. Graphs and statistical analyses were performed with GraphPad Prism 8.0 (GraphPad Software) and Excel (Microsoft). All the experiments were performed at least twice. Experiment sample numbers used for statistical testing were given in the corresponding figures and/or legends. Statistical comparison between two strains was performed by the two-tailed unpaired Student's t-test or χ^2 test as described in each figure legend, considering two-tailed *p*-values exceeding 0.05 to be not significant. We used this key for asterisk placeholders to indicate *p*-values in the figures: e.g., ****, $p < 0.0001$.

SUPPLEMENTAL REFERENCES

Moreno, S., Klar, A., and Nurse, P. (1991). Molecular genetic analysis of fission yeast *Schizosaccharomyces pombe*. Methods Enzymol *194*, 795-823.

Sato, M., Dhut, S., and Toda, T. (2005). New drug-resistant cassettes for gene disruption and epitope tagging in *Schizosaccharomyces pombe*. Yeast *22*, 583-591.
